# Scalable Decision Support at the Point of Care: A Substitutable Electronic Health Record App for Monitoring Medication Adherence

**DOI:** 10.2196/ijmr.2480

**Published:** 2013-07-22

**Authors:** William Bosl, Joshua Mandel, Magdalena Jonikas, Rachel Badovinac Ramoni, Isaac S Kohane, Kenneth D Mandl

**Affiliations:** ^1^Children's Hospital Informatics ProgramBoston Children's HospitalHarvard Medical SchoolBoston, MAUnited States; ^2^Department of Healthcare InformaticsUniversity of San FranciscoSan Francisco, CAUnited States; ^3^Children's Hospital Informatics ProgramBoston Children's HospitalBoston, MAUnited States; ^4^McKinsey & CompanyPalo Alto, CAUnited States; ^5^Harvard School of Dental MedicineBoston, MAUnited States

**Keywords:** electronic health record, personal electronic health record, hospital information systems, medical informatics applications, accountable care organizations, medication adherence

## Abstract

**Background:**

Non-adherence to prescribed medications is a serious health problem in the United States, costing an estimated $100 billion per year. While poor adherence should be addressable with point of care health information technology, integrating new solutions with existing electronic health records (EHR) systems require customization within each organization, which is difficult because of the monolithic software design of most EHR products.

**Objective:**

The objective of this study was to create a published algorithm for predicting medication adherence problems easily accessible at the point of care through a Web application that runs on the Substitutable Medical Apps, Reusuable Technologies (SMART) platform. The SMART platform is an emerging framework that enables EHR systems to behave as “iPhone like platforms” by exhibiting an application programming interface for easy addition and deletion of third party apps. The app is presented as a point of care solution to monitoring medication adherence as well as a sufficiently general, modular application that may serve as an example and template for other SMART apps.

**Methods:**

The widely used, open source Django framework was used together with the SMART platform to create the interoperable components of this app. Django uses Python as its core programming language. This allows statistical and mathematical modules to be created from a large array of Python numerical libraries and assembled together with the core app to create flexible and sophisticated EHR functionality. Algorithms that predict individual adherence are derived from a retrospective study of dispensed medication claims from a large private insurance plan. Patients’ prescription fill information is accessed through the SMART framework and the embedded algorithms compute adherence information, including predicted adherence one year after the first prescription fill. Open source graphing software is used to display patient medication information and the results of statistical prediction of future adherence on a clinician-facing Web interface.

**Results:**

The user interface allows the physician to quickly review all medications in a patient record for potential non-adherence problems. A gap-check and current medication possession ratio (MPR) threshold test are applied to all medications in the record to test for current non-adherence. Predictions of 1-year non-adherence are made for certain drug classes for which external data was available. Information is presented graphically to indicate present non-adherence, or predicted non-adherence at one year, based on early prescription fulfillment patterns. The MPR Monitor app is installed in the SMART reference container as the “MPR Monitor”, where it is publically available for use and testing. MPR is an acronym for Medication Possession Ratio, a commonly used measure of adherence to a prescribed medication regime. This app may be used as an example for creating additional functionality by replacing statistical and display algorithms with new code in a cycle of rapid prototyping and implementation or as a framework for a new SMART app.

**Conclusions:**

The MPR Monitor app is a useful pilot project for monitoring medication adherence. It also provides an example that integrates several open source software components, including the Python-based Django Web framework and python-based graphics, to build a SMART app that allows complex decision support methods to be encapsulated to enhance EHR functionality.

## Introduction

Non-adherence to prescribed medications is a serious health problem costing an estimated $100 billion per year in the United States [1]. Despite considerable research on poor medication adherence based on retrospective electronic health records (EHR) data analyses, there are few real-world implementations of systems that provide adherence related feedback to clinicians at the point of care [2]. While poor adherence should be addressable with point of care health information technology (IT), integrating new solutions with existing EHR systems require buy-in and customization within each organization. Such integration is made more difficult by the monolithic software design of the majority of EHR products [3].

The software application described in this paper was designed to operate within the Substitutable Medical Apps, Reusable Technology (SMART) framework. SMART [4] is funded by The Office of the National Coordinator for Health Information Technology through the Strategic Health IT Advanced Research Projects (SHARP) program. The goal of the SMART project is to specify an application programming interface to enable use of substitutable applications on EHRs—essentially reimagining EHRs as smartphone-like platforms that run apps [4-6].

**Figure 1:**
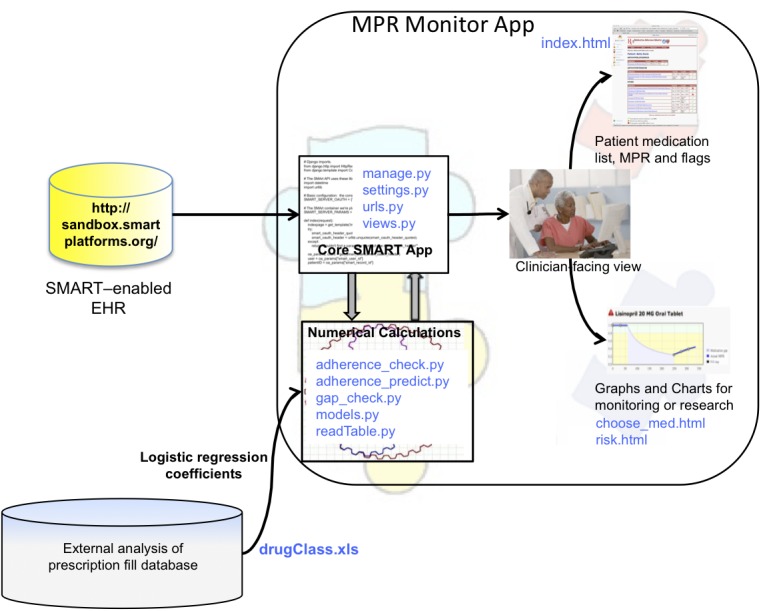
The SMART platform allows data to be accessed from any SMART-enabled electronic health record. The modular design illustrated here shows components in the MPR Monitor app. Each of the components attached to the core app may be modified without affecting the others. Additional components, such as new displays, may be added independently of the others. Actual file names from the application code are shown in blue letters in the figure.

The SMART Medication Possession Ratio (MPR) Monitor app implements a recently published medication adherence prediction algorithm [7], which can help identify poor adherers early. The app presents an interface to a physician with information about individual patients. It was designed to access patient prescription fulfillment information from a SMART-enabled EHR, which is currently represented by the SMART reference EHR [8]. Patient data obtained from the EHR is used as input to an adherence prescription algorithm to determine whether a patient is likely to be non-adherent after one year, based on data obtained within the first 60 to 120 days following the first prescription fill. The algorithm used to predict the likelihood of future adherence was derived independently of the MPR Monitor app by statistical modeling with data from a large national insurance prescription fulfillment dataset. In addition to one-year non-adherence predictions, other clinically useful data is computed and displayed. This includes a gap-check algorithm that determines if gaps greater than 30 days have occurred at any time during the prescription for each medication. The organization of the app is illustrated in Figure 1. The figure shows file names from the application code in blue letters in the figure to illustrate the modularity and also as a visual guide for those who would use this as an example for writing a SMART app. The files in the “Core SMART App” box are standard required files for any Django application. SMART-specific code is embedded in these. Codes for numerical simulation are pure python calculations and have no references to SMART or Django. The graphical displays are html and JavaScript, as needed by standard Django programs.

The goal of the MPR Monitor app is to present information to an authorized clinician in a graphical form that can be quickly reviewed to determine if additional action is required. The modular design of this app will allow alternate or additional computations with the prescription data that is obtained by the core app from the EHR, incorporation of new coefficients into the app when external data allows, or addition of new visual components to the physician view. In this way, the MPR Monitor can evolve in functionality with no changes to the EHR programming interface. Our implementation demonstrates an approach to building an application that delivers clinical decision support at the point of care using EHR data. The software design decisions discussed herein are intended to serve as an example and template for other applications that run on top of a SMART-enabled EHR.

## Methods

### Software Application Development

Several design decisions were made. Some are common to all Web application design, others specific to the SMART Application Program Interface (API). SMART Apps using the SMART API can be embedded within any SMART-enabled system, which is often an EHR system used by physicians, but might also be a personal health record used by patients, or a data-analytics platform such as [9] used by researchers. Patient data used for adherence monitoring must be marshaled by the SMART container, often an EHR, and exposed by a SMART API.

The SMART framework allows considerable flexibility for choosing programming language, frameworks, and support packages. A SMART app can access the SMART API using either simple Javascript calls from a client library or using standard OAuth authentication and REST (REpresentational State Transfer), a style of software architecture for distributed systems such as the World Wide Web) calls to a SMART container. Both of these approaches can be used to get at the same underlying data. The latter approach is appropriate for applications that may need to perform significant computational processing, such as when using additional external data sources as the MPR Monitor app does.

A SMART REST app may be written in any language with existing OAuth libraries. One such widely used language is Python. Since Python is a general purpose programming language with well-developed libraries for many data analysis tasks, including statistics, analytics, and graphical display, it was a versatile choice for building the MPR Monitor app. A commonly used Python framework for Web application development is Django. Although the server-side processing performed by the MPR Monitor app is relatively simple, we wanted to develop the application within a general framework like Django so that it may serve as an example for more complex app development.

The Django Web framework [10] uses Python as its core programming language. Other python-based Web frameworks exist, but Django is one of the most widely used in commercial applications and is capable of handling complex Web interface demands. We chose Django for the MPR Monitor because it will enable great flexibility for adding future functionality. It also enables the MPR Monitor app to be used by others as a general-purpose template for creating a wide range of new SMART apps.

Because the Django is written in Python, mathematical and statistical modules written with powerful Python libraries, as illustrated by the “embedded MPR calculation” box in Figure 1, are easily integrated into the framework. Statistical algorithms can be written entirely in Python code, which can also easily wrap code written in other languages such as R and C++. In this way, analytics can be embedded directly into the app to create flexible and sophisticated EHR functionality.

We emphasize that this medication adherence app is intended to be a useful tool monitoring medication adherence that can evolve over time, while providing a general example for app developers, particularly clinical researchers who confront real-world health IT challenges and may wish to quickly assemble apps for testing new ideas. The components made for monitoring non-adherence risk discussed below can easily be modified and assembled into new apps.

### Non-Adherence Checks

The MPR Monitor implements three different non-adherence checks. Two of these, the 30-day gap check and the actual MPR < 0.8 check, are indications of a current adherence problem. These two algorithms run on all prescription drugs in the medical record, regardless of drug class. Any medication found to be currently non-adherent is flagged using a bright red warning triangle. A third check uses a logistic regression model to predict non-adherence at one year, based on prescription fill information at 60, 90, or 120 days following the first fill. The goal of the prediction is to detect a potential non-adherence problem and alert the clinician before non-adherence becomes an actuality. The logistic regression is run on all medications that pass the 30-day gap detection test, but only for medications in the three drug classes for which regression models are currently available, which include antihyperlipidemics, antihypertensives, and oral hypoglycemics. A medication for which the patient is non-adherent is flagged with a yellow warning triangle.

### Algorithm Implementation

The origins of the MPR Monitor began with an approach to medication adherence that was based on monitoring prescription-filling behavior [11,12]. Although this approach was an indirect measure of adherence to a prescribed medication regime, patients who do not fill a prescription are unlikely to be taking the medication as prescribed. Thus, fulfillment histories provide a practical approach to detecting non-adherence. In a retrospective study of dispensed medications from a large private insurance plan, Jonikas and Mandl, analyzed over 8.5 million prescriptions and described an approach that predicted adherence problems one year from the first fulfillment by analyzing the initial 2 to 4 months of fulfillment data [7]. The primary variable predicted was the medication possession ratio (MPR), a measure of the number of days a patient possessed a prescribed medication (based on prescription fulfillment history) divided by the number of days since the first prescription fill:

MPR = (Number of fills * daily doses per fill) / (total days since first fill)

**Figure 2:**
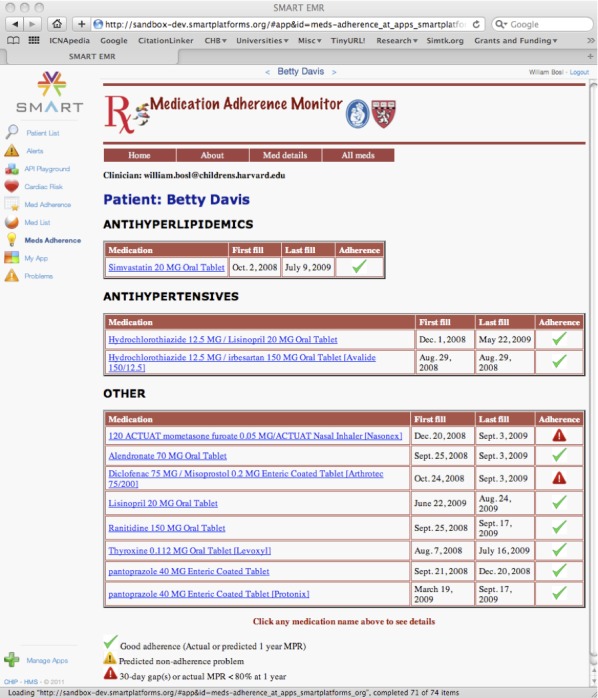
The first page displayed to the clinician for a single patient lists all the currently prescribed medications, grouped by categories for which logistic predictions are currently possible in the internal model. These groupings may be useful to the clinician beyond their use in non-adherence prediction. Easily viewed symbols are used to flag actual or predicted adherence problems.

**Figure 3:**
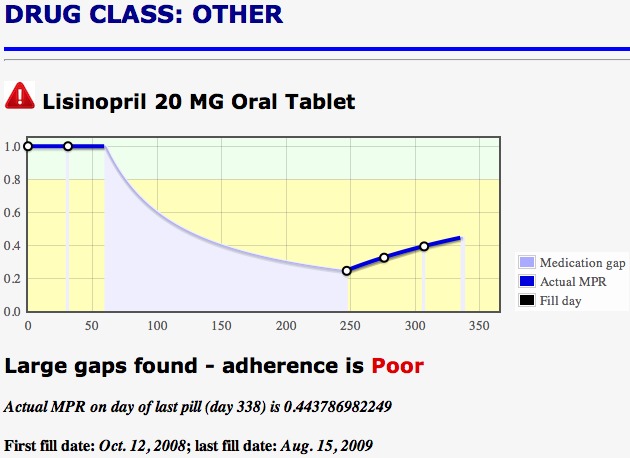
Detailed graphical information about a specific drug fulfillment history appears when the user clicks on any drug from the list shown in Figure 2.

Two tests were implemented for early identification of patients at risk for non-adherence. The first was a straightforward 30-day gap check used on every medication in the patient’s record. The gap check subtracts the number of daily medication doses from the elapsed time since the first fulfillment. If the gap is greater than 30 days, an alert is triggered. We consider this a more serious issue, since non-adherence is already detected and use a red warning symbol to visually alert the clinician. An example of the red warning triangle can be seen in Figures 2 and 3.

**Figure 4:**
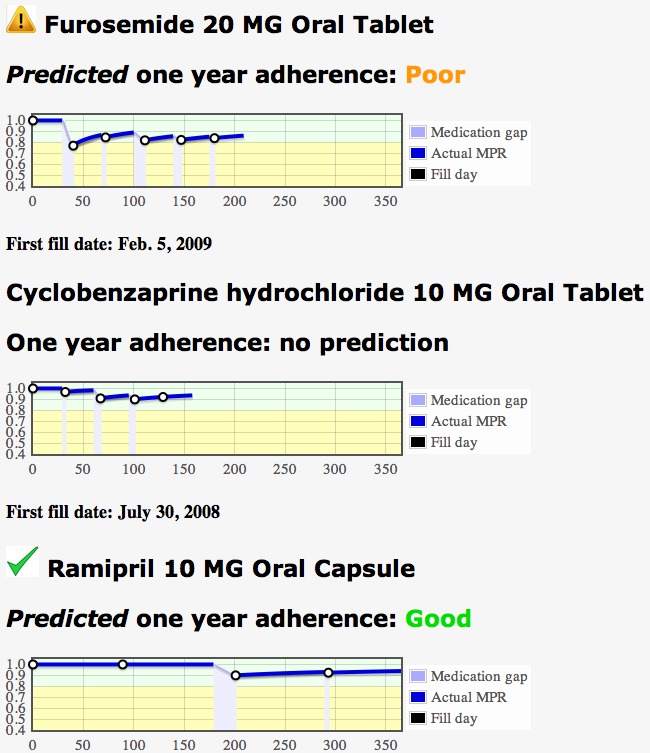
Detailed MPR information for each medication can be displayed in graphical form. Cyclobenzaprine is not in one of the classes for which a 1-year adherence prediction can be made. No current non-adherence issues are detected (gap check and low MPR check), so no warning flags are shown. A green check is not displayed because no future prediction can be made. The one-year MPR for Furosemide is predicted to be below 0.8 at one year in this case, based on patient information and MPR value at 120 days, even though the MPR value at 120 days and the latest date (200 days) is above the threshold. Prescription fill data for Ramipril is available for a full year, and the actual 1-year MPR is above threshold (0.8), so a green check is displayed.

A second test for non-adherence uses a logistic regression model with two independent input variables, the patient’s age and the MPR value at the time of the prediction, to predict a binary outcome, acceptable or poor adherence, one year after the first fulfillment. The logistic regression parameters were determined separately for three classes of drugs: antihyperlipidemics, antihypertensives, and oral hypoglycemics. These drug classes were selected because improved health outcomes for these chronic medications require consistent adherence over time [13-16]. Models to predict MPR value at one year from the first prescription fill were computed at 60, 90, and 120 days from the first fill for each of the three different drug classes. The model description and coefficients were published in the free-access paper [7], yielding publicly accessible, quantitative research results. The MPR app demonstrates a way to embed these results in a clinical decision support tool that may be interoperable with existing EHR systems. Since future *predicted* non-adherence might be considered less serious than actual non-adherence, a yellow warning triangle is used to visually signal this adherence problem, as illustrated in Figure 4.

The second drug in Figure 4, cyclobenzaprine hydrochloride, is typically used as a muscle relaxant to relieve acute skeletal muscle pain or injury. No predictive modeling was done for this class of medications, thus predicted non-adherence is not possible. Importantly, however, if the type of logistic regression coefficients derived for the three drug classes previously described [7] becomes available for this or any other drug classes, the coefficients can easily be added to an external text file that contains this information for the app, thus extending its capability. Because the SMART app was designed to be interoperable with any SMART-enabled EHR, such an extension will not affect or require any changes to the way the app operates with these databases.

The MPR can easily be computed at every time for which medication data for a patient is available and may be used in an additional test for non-adherence. A common indication of non-adherence is when the MPR is below 0.8. Thus for any prescription medication in the EHR, if the MPR is less than 0.8 at the latest time in the record, it is considered a non-adherence indication and is flagged with a red triangle. It is no longer a predicted, future non-adherence issue, but a present condition. A graphical display of the MPR values may be selected so that a clinician can visually see when low MPR values or gaps occur throughout the prescription history. This is illustrated in Figures 3 and 4.

## Results

### The MPR Monitor App

Currently, the MPR Monitor app is designed for use by a physician or health care provider to quickly review all medications in a patient record for potential non-adherence. The initial app screen shown once a patient has been chosen is presented in Figure 2. All medications are listed, along with the date of first prescription fill, last fill, and a color-coded symbol that visually indicates adherence level: a green check for adherence, a red warning symbol for current non-adherence (current MPR < 0.8 or a 30-day gap), and a yellow warning symbol for predicted non-adherence if the regression model has determined that non-adherence is likely at 1 year, based on model predictions from the latest MPR available from 60, 90, or 120 days after the first fill.

If more information about the fulfillment history for a particular medication is desired, the user may click on any medication listed and a plot like that shown in Figure 3 will appear. This page shows visually if a patient possesses medication based on the last fill and current date and assumes that the patient is taking the medication. After all doses have run out, if there has been no succeeding fulfillment, a gap is indicated by a break in the MPR line. The actual value of the MPR is calculated and shown for every data point. In this way, the health care provider can visualize an entire year of fulfillment history for the medication. It may also be useful to see all medication fulfillment graphs of a patient at one time. This can be requested by clicking the “All Med” button at the top of any page. Plots such as those in Figure 3 appear for all medications. The plots and simple adherence indicators enable health care providers to quickly see medication adherence problems for any patient. Colors, symbols, and shading were used to make visual interpretation of the plot immediate, once the caregiver becomes familiar with the app. Additional detailed information is included in text or numerical form, such as exact MPR values, fulfillment dates, and dosage.

Gaps are easily visualized as gray dropout regions. The running MPR value is shown as a bright blue line. Prescription fill events are shown as circles on the graph. Mouse-over the graph lines will reveal a pop-up display with precise day and MPR values at any point on the graph. Non-adherence predictions are not made for medications that do not belong to one of the drug classes for which the regression model was designed. However, the current adherence in the case illustrated here is poor, based on a 196-day gap. The gap check is done for all prescription medications in the medical record. The required model inputs that are automatically accessed by the app are patient age at time of first fulfillment, the MPR on the prediction day, and the prediction day, either 60, 90, or 120 days since the first fill. This information is either read directly from the EHR (patient age) or derived from information contained in the record (prescription fulfillment history).

The analytics implemented in this app are relatively simple, but illustrate an important principle of reusable technologies: new apps directed at different clinical settings and diseases can be assembled quickly by adopting components from other apps. The core SMART app, the “embedded” calculations component, and the clinician-facing displays are all designed to be reusable components.

Users may run the application remotely using the SMART development sandbox, which can be accessed at [8]. Links to the source code repository may be found on GitHub [17]. The software is listed as the MPR Monitor and can be found either directly on GitHub or through the SMART website listed above.

### Interface Functionality and Design

Designing a Web interface requires considerable attention to the needs of the client–in this case a busy clinician who will review the medication fulfillment information for each patient. In a clinical setting, we felt that the most important information needed was a complete listing of all the medications that a patient was prescribed, together with a clear and obvious adherence status symbol for each medication. A green check mark indicated adequate adherence, while a yellow or red triangle, a universal warning symbol, signaled an adherence problem requiring attention. We chose to differentiate actual adherence problems (signified by a 30-day gap in medication or an MPR value below 0.8) and predicted adherence problems (where the MPR is greater than 0.8 but is predicted by the app to fall below 0.8 at the 1-year mark). Although the internal criteria for determining adherence or non-adherence might change in future implementations of the app as more data is incorporated into the predictive model, the meaning and use of the bright colored symbols should remain the same.

If a physician desires more information about a specific medication, clicking on the drug name will bring up a graphical display and the MPR values of a patient for a specific prescription over time, generated by Flot. Flot is an open source graphing package that uses the JavaScript plotting library for jQuery, with a focus on simple usage, professional appearance, and interactive features. The Flot package is self-contained in the code distribution of MPR Monitor app. Calls to Flot functions are embedded within the html interfaces found in the standard Django “template” directory. Flot enabled a few interactive features to be built into the app, such as the ability to point the cursor to any point on a graph of the patient’s MPR curve over time and read the numerical value at that time. This interactive feature can be tested on the SMART sandbox. Shading and colors make it visually obvious where adherence was determined to be a concern, according to the adherence criteria built into the app.

In the design of the MPR Monitor app, drug class information was read from an external file, packaged locally with the application in a simple text file. A separate text file contains the regression model coefficients together with the applicable drug class and patient age. This enables the app to be updated with new information in the future if further regression models are tested and become available. The app can thus evolve without new programming or app installation by supplying a configuration file with updated model data.

The SMART MPR Monitor app is currently installed in the SMART reference container (a skeletal EHR with the SMART application programming interface) where it can be used and tested. General information about SMART apps is available on the SMART project home page [4], where which users can create an account and run installed apps on the SMART reference container. Once a new account is created, users can go to the sandbox [8], load the MPR Monitor, and run the app on sample patients provided in the sandbox.

## Discussion

### Principal Findings

We have developed a modular application that detects medication non-adherence, is easily maintained and upgraded as new research or data becomes available, and has sufficient generality to be used as an example for other SMART apps. The design choices were made to enable open source statistical, graphical, and decision support Python packages to be assembled in a SMART-enabled app. Software can be downloaded and modified.

The need for informatics tools that monitor non-adherence to prescriptions is representative of a more general emerging need for more sophisticated data analysis tools that can access, integrate, and find diagnostic information in a wide variety of patient data types. Physicians have always needed to be skilled at gathering diverse sources of data from examinations and tests, synthesizing the data into a diagnosis, and determining a therapeutic strategy based on current medical knowledge and the patient’s particular circumstances. However, advances in medical technology are producing enormous quantities of data that introduce new challenges to the physician for integrating all the information into clinical decision making. As EHRs are deployed widely to collect and manage medical data, and thus help to control costs, improve coordination of care, and eliminate errors, flexible integration of software applications with the EHR would extend their capabilities, and also create a broad market for apps.

In its current form, the MPR Monitor has a modular architecture that includes a clinician-facing interface, statistical routines that compute medication adherence information such as the MPR, and graphical display webpages that report information to the clinician. These can be easily modified or new modules appended to quickly assemble new apps that utilize information from any SMART-enabled EHR system with custom or reusable analytics.

### Conclusions

Non-adherence to prescribed medications has many potential causes. Patients may not be able to afford the medications, while some may simply forget or confuse multiple drugs. Others may not understand the importance of continuing a regiment even if they feel better before the regiment ends. Regardless of the cause, the first step in solving the problem is to identify patients who are non-adherent, or those who are likely to be non-adherent before the problem arises.

The SMART API enables ready diffusion of software code and algorithms in the form of apps that can interact with any EHR that has been modified to support the SMART interoperability standard. The MPR Monitor presented illustrates how a SMART application can be implemented in practice. Because SMART apps are readily substituted, future similar apps with improved features, or better evidence-based methods can readily replace the existing versions. The community of users can then determine which apps will be adopted and which will fade away from disuse, just as the value of commercial apps is decided.

## Abbreviations

API: application program interface
EHR: electronic health record.
IT: information technology
MPR: Medication Possession Ratio
SHARP: Strategic Health IT Advanced Research Projects
SMART: Substitutable Medical Apps, Reusable Technology
